# SCF Ensures Meiotic Chromosome Segregation Through a Resolution of Meiotic Recombination Intermediates

**DOI:** 10.1371/journal.pone.0030622

**Published:** 2012-01-23

**Authors:** Shin-ya Okamoto, Masamitsu Sato, Takashi Toda, Masayuki Yamamoto

**Affiliations:** 1 Department of Biophysics and Biochemistry, Graduate School of Science, University of Tokyo, Hongo, Tokyo, Japan; 2 PRESTO, Japan Science and Technology Agency, Honcho Kawaguchi, Saitama, Japan; 3 Laboratory of Cell Regulation, Cancer Research UK, London Research Institute, Lincoln's Inn Fields Laboratories, London, United Kingdom; 4 Kazusa DNA Research Institute, Kisarazu, Chiba, Japan; National Cancer Institute, United States of America

## Abstract

The SCF (Skp1-Cul1-F-box) complex contributes to a variety of cellular events including meiotic cell cycle control, but its function during meiosis is not understood well. Here we describe a novel function of SCF/Skp1 in meiotic recombination and subsequent chromosome segregation. The *skp1* temperature-sensitive mutant exhibited abnormal distribution of spindle microtubules in meiosis II, which turned out to originate from abnormal bending of the spindle in meiosis I. Bent spindles were reported in mitosis of this mutant, but it remained unknown how SCF could affect spindle morphology. We found that the meiotic bent spindle in *skp1* cells was due to a hypertension generated by chromosome entanglement. The spindle bending was suppressed by inhibiting double strand break (DSB) formation, indicating that the entanglement was generated by the meiotic recombination machinery. Consistently, Rhp51/Rad51-Rad22/Rad52 foci persisted until meiosis I in *skp1* cells, proving accumulation of recombination intermediates. Intriguingly bent spindles were also observed in the mutant of Fbh1, an F-box protein containing the DNA helicase domain, which is involved in meiotic recombination. Genetic evidence suggested its cooperation with SCF/Skp1. Thus, SCF/Skp1 together with Fbh1 is likely to function in the resolution of meiotic recombination intermediates, thereby ensuring proper chromosome segregation.

## Introduction

SCF (Skp1-Cullin 1-F-box) complexes constitute a ubiquitin ligase family, members of which are involved in ubiquitylation and degradation of a number of cellular factors including cell cycle regulators and transcription factors. SCF complexes are composed of an unaltered core of Cullin 1, the RING finger protein Rbx1/Roc1/Hrt1, and Skp1, which can bind a number of different F-box proteins. F-box proteins act as receptors for specific substrates for ubiquitylation. The fission yeast *Schizosaccharomyces pombe* has eighteen F-box proteins, according to the *S. pombe* gene database (GeneDB; www.genedb.org/genedb/pombe/), which may regulate various cellular processes. It has been shown that F-box proteins Pop1 and Pop2 are required for the maintenance of genome ploidy through the timely destruction of Rum1 and Cdc18, which are a CKI (cyclin-dependent kinase inhibitor) and a DNA replication factor, [1], [2], [3], [4]. Another F-box protein Pof1 regulates the cadmium response by targeting the transcription factor Zip1, which controls expression of cadmium-induced genes [5]. Pof3, an F-box protein also, is involved in the maintenance of genomic integrity together with its binding partner Mcl1 [6], [7]. Phenotypes of the *pof3*-deletion (*pof3*Δ) mutant displays defects in genome integrity such as shortened telomeres, and the Rad3-dependent DNA damage checkpoint is indispensable for the survival of *pof3*Δ cells [7]. Pof3 is involved in the downregulation of the GATA-type transcription factor Ams2, which activates transcription of the core histone genes during S phase [8]. Further, Pof6 is required for cell separation [9], with its binding partner Sip1 [10]. Fbh1, an F-box protein containing a DNA helicase domain, is involved in the processing of chromosomal recombination intermediates [11].

Mutants of Skp1 are known to exhibit defective phenotypes in the cell cycle control. The *skp1-a7* temperature-sensitive mutant arrests in G2 phase of the mitotic cell cycle due to activation of the Rad3-dependent DNA damage checkpoint [12]. The *rad3*Δ *skp1-a7* double mutant suppressed the G2 arrest at the high temperature. This indicates that the DNA damage checkpoint is ectopically activated in the *skp1-a7* mutant [12]. Interestingly, the *skp1-a7* mutant exhibits abnormally-bent spindles and fails in nuclear division at anaphase [13]. This implies that Skp1 may regulate organization of spindle microtubules and/or elasticity of the nuclear envelope. The *rad3*Δ *skp1-a7* double mutant, however, no longer shows the bent-spindle phenotype in anaphase [13]. The F-box protein responsible for this bent-spindle phenotype has not been identified.

Thus, fission yeast Skp1 regulates a wide variety of cellular events in the mitotic cell cycle, cooperating with many F-box proteins, but little is known about its function in meiosis. To investigate the possible commitment of Skp1 to meiosis, we observed the behavior of the *skp1* mutant in meiosis and subsequent sporulation. We noticed that abnormal spores were generated in the *skp1-a7* mutant. We also realized that *skp1-a7* cells frequently displayed abnormal X-shaped spindles in meiosis II and failed in nuclear division. Further analyses revealed that these phenotypes originate from the bent spindle generated in meiosis I, which is similar to the one previously observed in mitosis. We show that phenotypic abnormalities observed in *skp*1 mutant cells are attributable to defects in Fbh1-mediated meiotic recombination.

## Results

**Figure 1:**
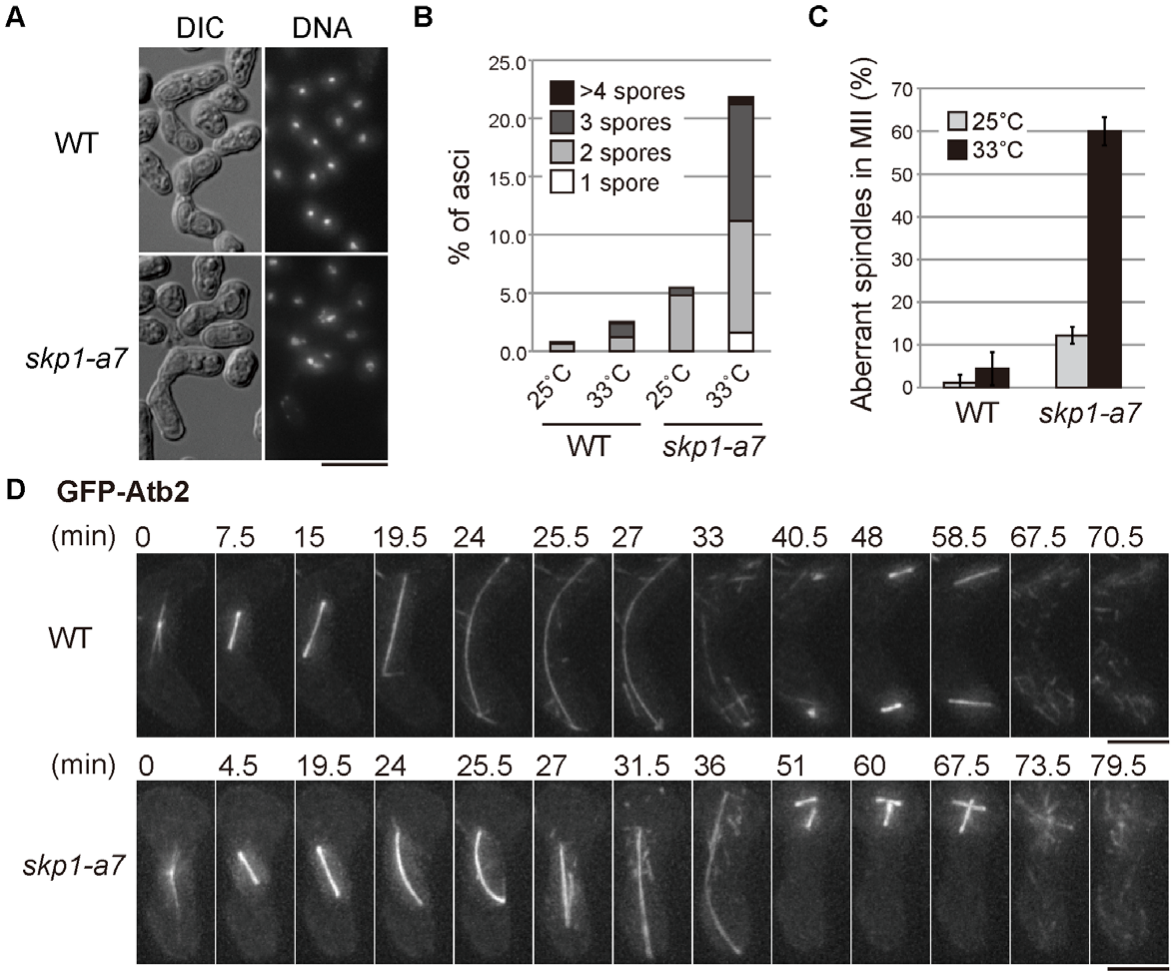
The *skp1-a7* mutant shows the bent spindle in MI. (**A**) Meiosis and sporulation were induced for wild-type (WT) and the *skp1-a7* strains at 33°C, the semi-restrictive temperature. Images of DIC and DNA stained with DAPI are shown. (**B**) The number of spores in WT and *skp1-a7* asci were counted. Percentages of aberrant asci only are shown in the graph. Most of WT cells generated four spores, whereas *skp1-a7* cells produced abnormal asci with 1∼3 and ≥5 spores. Meiosis was induced at the indicated temperature. *n* = 250. (**C**) Percentages of aberrant ‘crossing’ spindles in MII. Meiosis was induced at the indicated temperature. *n*≥30. (**D**) Time-lapse images of WT and *skp1-a7* cells expressing GFP-Atb2 (α2-tubulin) from the MI onset until anaphase/telophase of MII. In the *skp1-a7* cell, the spindle abnormally bent at 25.5 min before full elongation, and collapsed at 27 min. Note that the WT spindle never bent before reaching the cell cortex. The crossing spindles in MII are shown at 51∼67.5 min. Bars, 5 µm.

To investigate the meiotic function of SCF/Skp1 in fission yeast, meiosis was induced in the *skp1-a7* mutant [12]. Throughout this study we performed observation of meiotic progression at the semi-restrictive temperature for the mutant (33°C), not at the restrictive temperature (36°C), because meiosis is intrinsically sensitive to high temperature and does not proceed at 36°C. After conjugation, wild-type zygotes underwent meiosis I (MI) and meiosis II (MII) and generated four spores in an ascus (Fig. 1A). In contrast, *skp1-a7* zygotes frequently produced an abnormal number of chromosome masses (less than four) and showed a high rate of two- or three-spored ascus formation (Fig. 1A,B). To pinpoint which stage of the cell cycle was impaired by the loss of functional Skp1, microtubules were visualized using GFP-Atb2 (green fluorescent protein-fused α2-tubulin). 14–15 hours after the induction of meiosis, we frequently found zygotes with abnormal spindle structure in MII. Most of wild-type zygotes (WT) showed a pair of spindles at MII, whilst spindles in about 60% of *skp1-a7* zygotes appeared to be abnormal, represented typically by a ‘cross’ of two spindles as shown in Fig. 1C.

Time-lapse imaging of WT and *skp1-a7* meiotic cells was performed to elucidate the reason for the spindle abnormality. In WT zygotes, a spindle elongated in straight in anaphase I until both ends of the spindle reached to the cell tips (24 min, top; Fig. 1D). Then the spindle bent slightly in accordance with the shape of the zygote and disassembled upon MI exit (33 min). After the completion of MI, an MII spindle started to form in each of the two nuclei (40.5 min) and elongated. In the *skp1-a7* mutant, the spindle morphology was almost normal in the early stage of MI (bottom; Fig. 1D). The spindle, however, did not fully elongate even nearly 20 minutes after the MI onset (19.5 min, Fig. 1D). Interestingly, the spindle started to bend without touching the cell cortex (24–25.5 min), and then collapsed (the V-shape spindle at 27 min), implying that an unusual intolerable tension was imposed to the spindle. After the collapse, two bundles were merged into one and resumed to grow to a comparable length to the WT spindle (31.5 and 36 min). This rebuilt spindle cannot be bipolar: two spindle poles are located only to one side of the broken spindle, and the other side (the broken ends of microtubules) has no SPBs. This unequal segregation of SPBs then caused the co-existence of two spindles in one nucleus in MII (48 min). We conclude that the collapse of an abnormally-bent spindle is the cause for the ‘crossing spindle’ observed in *skp1-a7* cells.

The bent spindle was observed previously during mitosis of the *skp1-a7* mutant [12], but the reason remained unclear. We thus sought for the reason to generate a bent spindle. One possibility might be that the spindle is hyperstabilized in the *skp1-a7* mutant. Alternatively, the architecture of the nuclear envelope might be altered. However, we could not obtain supporting data for these hypotheses (data not shown). We then hypothesized that the abnormal tension might be caused by some event(s) specifically seen before or during MI, because the MII spindle of the *skp1-a7* mutant was not bent (51, 60 and 67.5 min, Fig. 1D). MI is characterized by the unique chromosome organization: homologous chromosomes derived from the parents are paired and form chiasmata, and then they are segregated (reductional division), in contrast to the separation of the sister-chromatids in mitosis and MII (equational division; reviewed in [14], [15]).

**Figure 2:**
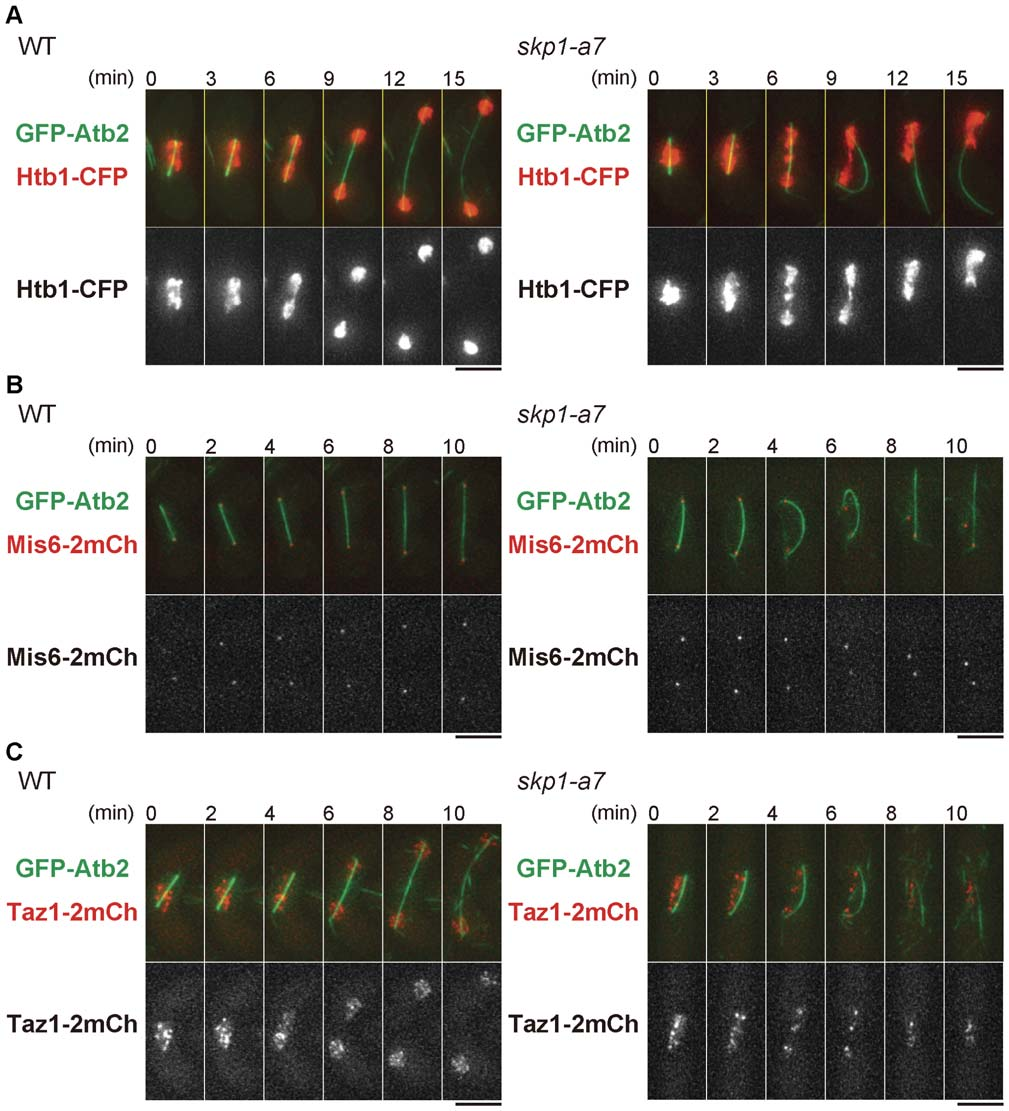
Chromosome segregation defects in *skp1-a7* cells at MI. Time-lapse images of WT and *skp1-a7* zygotes at MI with indicated fluorescent proteins filmed at 33°C. (**A**) GFP-Atb2 for microtubules (green) and Htb1-CFP for histone H2B (red). In *skp1-a7*, the chromosome bridge is seen at 6∼9 min, followed by an appearance of the bent spindle evident at 9 min. The spindle then collapsed and the rebuilt spindle is seen at 12∼15 min. (**B**) GFP-Atb2 (green) with Mis6-2mCh for kinetochores (red). (**C**) GFP-Atb2 (green) with Taz1-2mCh for telomeres (red). Bars, 5 µm.

**Figure 3:**
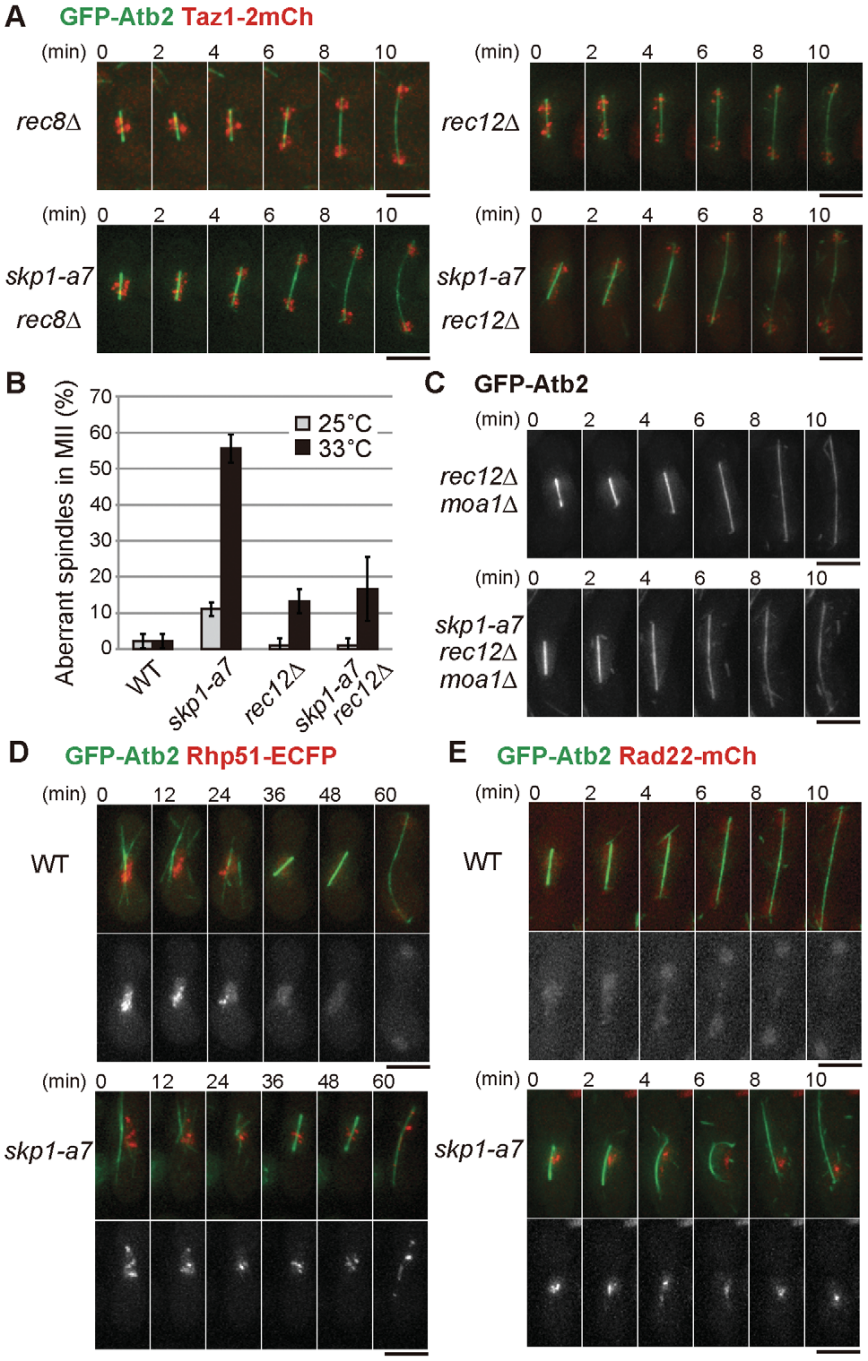
The chromosome entanglement is made through meiotic recombination. (**A**) Chromosome segregation at MI in WT and *skp1-a7* cells was monitored in the *rec8*Δ and *rec12*Δ background. Note that the spindle was not bent in the *skp1-a7 rec8*Δ and *skp1-a7 rec12*Δ. GFP-Atb2 (green) and Taz1-2mCherry (red) are shown. (**B**) Suppression of the crossing-spindle phenotype in *skp1-a7* cells by *rec12*Δ. Frequency of ‘crossing’ spindles in MII at 25°C and 33°C. *n*≥30. Error bars, SEM. (**C**) The spindle was straight in *skp1-a7 rec12*Δ *moa1*Δ zygotes. Note that in *rec12*Δ *moa1*Δ cells, sister-chromatids are joint until metaphase I and then the sister-chromatids are segregated because of the kinetochore bipolarity. (**D**) Rhp51-ECFP foci persisted until MI in the *skp1-a7* mutant. GFP-Atb2 (green) and Rhp51-ECFP (red) are shown. (**E**) Rad22-mCherry foci also persisted until MI in the *skp1-a7* mutant. Bars, 5 µm.

The behavior of chromosomes in the *skp1-a7* mutant was monitored using Htb1-CFP, histone H2B fused with cyan fluorescent protein, together with GFP-Atb2 to visualize microtubules. In MI of WT cells, Htb1-CFP segregated in equal amounts (Fig. 2A). In the *skp1-a7* mutant, however, an unsegregated bulge of Htb1-CFP was seen before the spindle started to bend (6 min, *skp1-a7*). Then the spindle bent, as if it were a bow with a series of ‘bridged’ chromosomes as a bowstring. When the spindle collapsed, most of the chromosome mass gathered to one side of the zygote (12 min), resulted in missegregation of the chromosomes. We then tracked the behavior of kinetochores by visulalizing Mis6 [16] tagged with two copies of mCherry. In WT, Mis6-2mCherry foci segregated equally in MI anaphase (anaphase I) (WT, Fig. 2B). In the *skp1-a7* mutant, Mis6-2mCherry foci split equally and reached to SPBs in anaphase I as in WT (0 min, *skp1-a7*), but the two foci moved back close to each other when the spindle bent and collapsed (6–10 min). This indicates that the kinetochore-microtubule attachment and kinetochore segregation in MI once occurred normally in the *skp1-a7* mutant, by the time the spindle collapsed. Telomeres, the ends of chromosomes, were next visualized using the telomere-binding protein Taz1-2mCherry [17]. In WT, Taz1-2mCherry foci segregated equally as anaphase I proceeded (Fig. 2C). In *skp1-a7*, however, some Taz1-2mCherry foci remained in the middle of the nucleus when the spindle bent (4, 6 min). Taking these observations together, the centromeric region of chromosomes appeared to separate normally but the arm region was not fully segregated at the anaphase I onset in the *skp1-a7* mutant. This was a likely cause of the chromosomal entanglement observed when the spindle bent. To confirm that the defects in arm separation and the emergence of the bent spindle are linked, we removed chromosome cohesion by deleting the *rec8* gene. Rec8 protein is a meiotic cohesin, which adheres sister and homologous chromosomes until metaphase I [18]. Upon the anaphase I onset, Rec8 is cleaved by separase and homologous chromosomes are segregated. Cells with *rec8* disrupted (*rec8*Δ) lose chromosomal cohesion in meiosis. Importantly, in the double mutant of *skp1-a7 rec8*Δ, the bent-spindle phenotype was rarely observed (Fig. 3A). This strongly indicates that chromosomes cannot be completely resolved in *skp1-a7* cells. As a consequence, the spindle might induce an intolerable tension that causes the sudden collapse.

The chromosomal non-disjuntion can be possibly due to meiotic recombination defects. To test this, we removed Rec12 from the *skp1-a7* mutant. Rec12 is a fission yeast ortholog of the Spo11 endonuclease, which induces double-strand breaks (DSBs) in chromosomes during meiotic prophase [19], [20]. In *rec12*Δ cells, homologous chromosomes are not recombined and hence do not form chiasmata [20]. The bent-spindle phenotype of *skp1-a7* cells was suppressed by introducing *rec12*Δ (Fig. 3A,B), indicating that the recombination of homologous chromosomes is involved in production of the chromosome non-disjunction observed in the *skp1-a7* mutant. The suppression was similarly seen in *rec12*Δ *moa1*Δ *skp1-a7* cells (Fig. 3C), in which the chromosome segregation is performed in an equational manner due to lack of kinetochore monopolarity [21]. This indicates that the non-disjunction does not occur among sister chromatids, supporting the possibility of the recombination-dependent entanglement.

We then visualized the DNA repair protein Rhp51 (the fission yeast ortholog of Rad51/RecA [22], [23]). Rhp51 binds to single- and double-strand DNA and promotes annealing and exchange of strands via its recombinase activity, and nuclear Rhp51 foci are markers of recombination intermediates that contain single-strand DNA (ssDNA) [24]. Rhp51-ECFP formed intense foci in the WT nucleus of meiotic prophase, which diminished as the cells entered MI (WT, Fig. 3D). By contrast, in *skp1-a7* zygotes, the Rhp51-ECFP foci persisted even during MI, when the cells displayed chromosome non-disjunction (*skp1-a7*, Fig. 3D). Rad22, another repair protein that interacts with Rhp51, also forms foci at DSB sites [25], [26]. *skp1-a7* zygotes exhibited prolonged localization of Rad22-mCherry foci even in MI (Fig. 3E). Persistence of Rhp51 and Rad22 foci indicates that the recombination complex, comprising the invading ssDNA and the recombination factors, was not properly processed. The persistence of the complex may cause the significant non-disjunction of chromosome arms.

**Figure 4:**
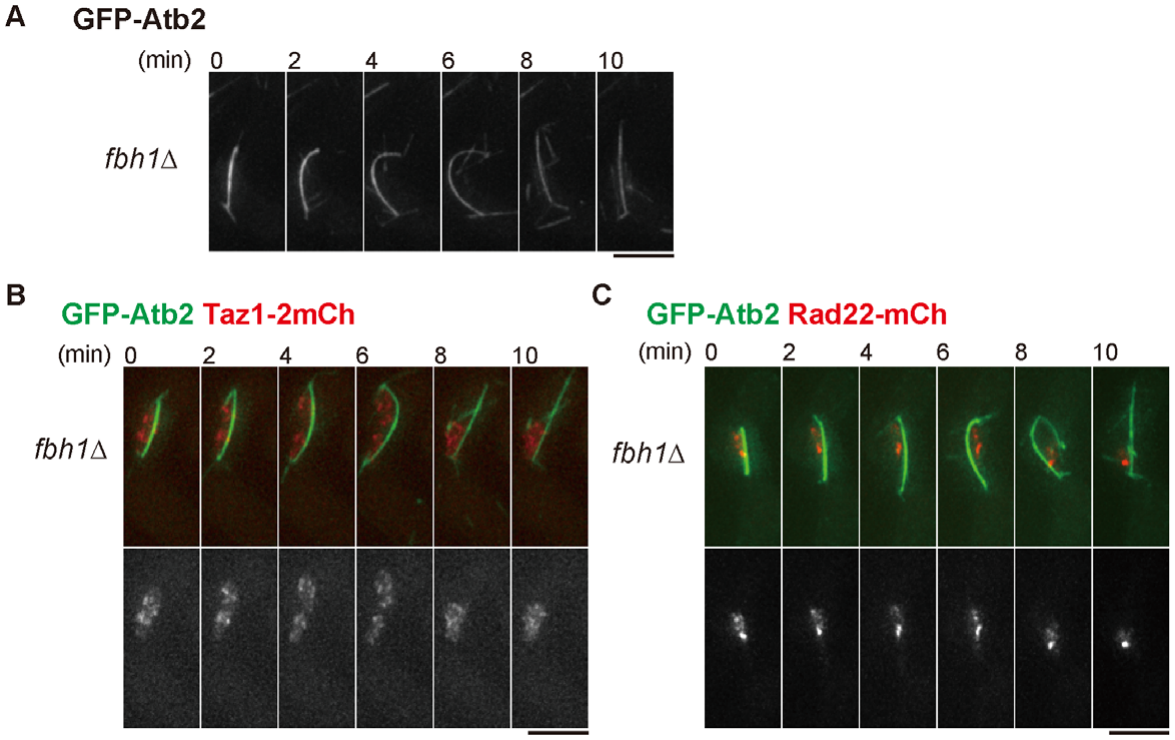
The *fbh1* deletion mutant shows similar phenotypes to the *skp1-a7* mutant. (**A**) The bent spindle was frequently observed in *fbh1*Δ cells at MI. Time-lapse imaging for GFP-Atb2 is shown. (**B**) The *fbh1*Δ mutant exhibited non-disjunction of chromosome arms visualized by Taz1-2mCherry (red) with GFP-Atb2 (green). (**C**) The *fbh1*Δ mutant showed persistent Rad22-mCherry foci until MI. Bars, 5 µm.

**Figure 5:**
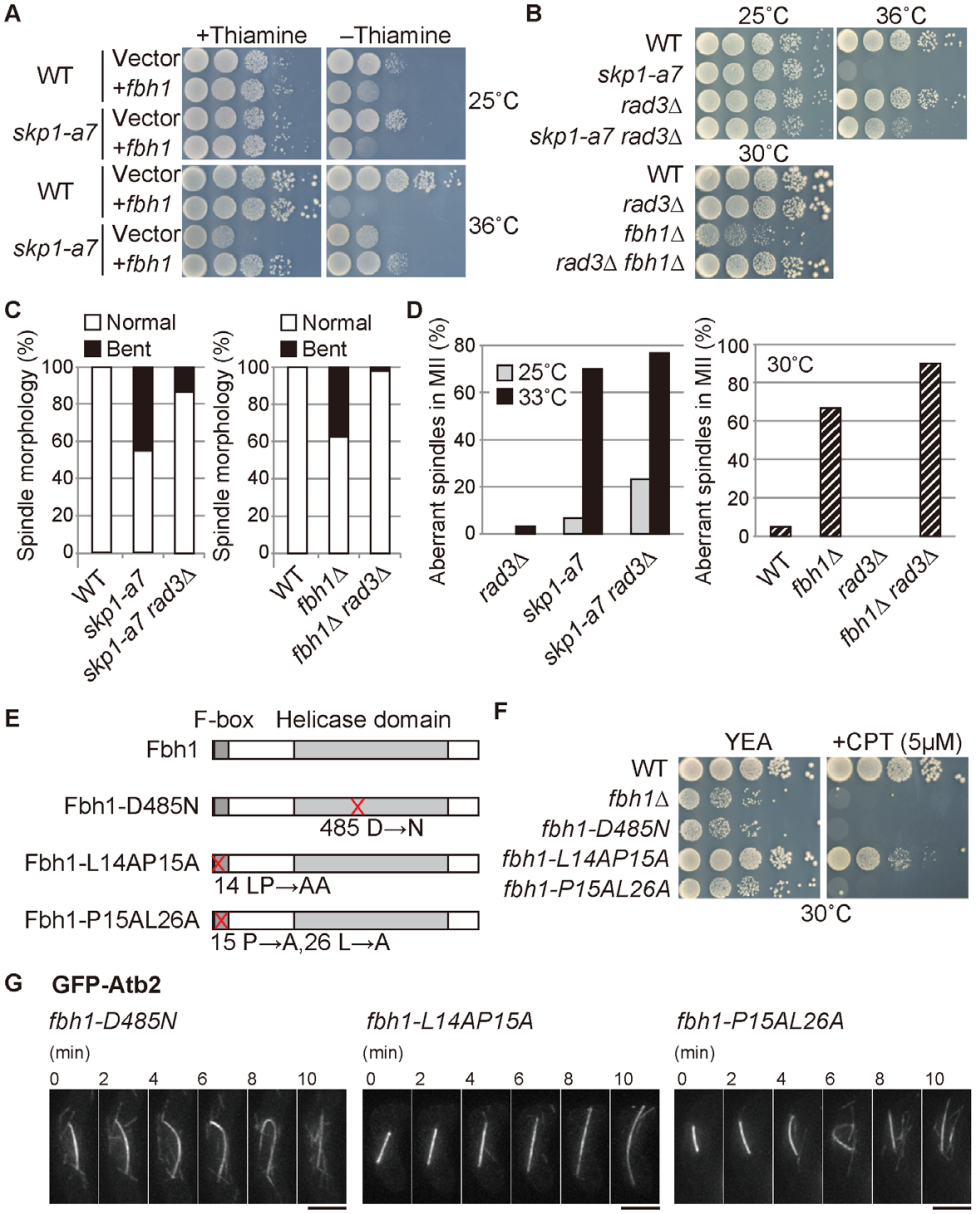
Skp1 together with Fbh1 resolves meiotic recombination intermediates. (**A**) Temperature sensitivity of the *skp1-a7* mutant was partially suppressed by overexpression of Fbh1 from the plasmid vector pREP1. Ten-fold serial dilution of the indicated strains was spotted on minimal media with or without thiamine and then incubated at 25°C and 36°C. Overexpression is induced on the medium without thiamine (−thiamine). (**B**) Growth defects of *skp1-a7* and *fbh1*Δ mutants were suppressed by *rad3*Δ, removal of the Rad3-dependent DNA damage checkpoint. (**C, D**) Frequency of the normal and bent spindle in mitosis (C) and the aberrant ‘crossing’ spindles in MII (D) in the indicated strains. *n*≥30. (**E**) Schematic diagram for Fbh1 WT and Fbh1-D485N (the helicase-dead form), Fbh1-L14A P15A and Fbh1-P15A L26A (the F-box-deficient form) mutant proteins. (**F**) Sensitivity to 5 µM CPT (camptothecin) was tested for the indicated strains. (**G**) *fbh1-D485N* and *fbh1-P15A L26A* mutants exhibited the bent-spindle phenotype, whereas *fbh1-L14A P15A* did not. GFP-Atb2 in MI of the indicated strains is shown. Bars, 5 µm.

SCF functions as a complex of Skp1, Cullin 1 and F-box proteins, which are thought to determine the specificity of binding proteins or degradation substrates [27]. We next sought for an F-box protein responsible for the meiotic function of SCF/Skp1 among eighteen F-box proteins reported to date (GeneDB; www.genedb.org/genedb/pombe/). Among deletion mutants of these F-box protein, we screened all viable strains for the one that reproduced the bent spindle seen in the *skp1-a7* mutant. None showed a bent-spindle in MI, except for *fbh1*Δ (Fig. 4A). Fbh1/Fdh1 is a unique F-box protein, which has an UvrD/REP helicase domain at the C-terminus, in addition to the conserved F-box motif at the N-terminus [11], [28]. Fbh1 is known to process the recombination intermediates in mitosis [11] and meiosis [29]. The frequency of the appearance of a bent spindle at MI in the *fbh1*Δ mutant reached almost 40% (see Fig. 5C), which was comparable to the percentage in the *skp1-a7* mutant. Similarly to *skp1-a7*, *fbh1*Δ cells showed defects in chromosome disjunction (indicated by Taz1-2mCherry, Fig. 4B). *fbh1*Δ cells also exhibited persistence of Rad22-mCherry foci during MI, like *skp1-a7* cells (Fig. 4C). Thus, the *fbh1*Δ mutant displayed nearly the same phenotype as the *skp1-a7* mutant during meiosis.

To further investigate if the phenotypes of *skp1-a7* cells could be explained by a lack of Fbh1 as the responsible F-box protein, the genetic interaction of *skp1* and *fbh1* was then tested. Elevated expression of Fbh1 partially suppressed the growth defects of *skp1-a7* at the restrictive temperature (Fig. 5A). As reported previously [12], the temperature sensitivity of the *skp1-a7* mutant was suppressed by removal of Rad3, the ATR kinase of the DNA damage checkpoint machinery (Fig. 5B). Deletion of the *rad3* ^+^ gene also suppressed the growth defects of the *fbh1*Δ mutant (Fig. 5B). These results prove the relevance of the *skp1-a7* and *fbh1*Δ phenotypes.

In mitosis, the bent-spindle phenotype was no longer observed in either the *skp1-a7 rad3*Δ or the *fbh1*Δ *rad3*Δ mutants (Fig. 5C) [13]. This could mean that the Rad3-dependent DNA damage checkpoint was ectopically activated in *skp1-a7* and *fbh1*Δ cells, which caused the defects [12]. Interestingly, however, removal of Rad3 did not suppress the ‘crossing’ spindles in MII (Fig. 5D). This suggests that the bent-spindle in MI was caused by persistent recombination intermediates generated in the process of meiotic recombination, but independently of the Rad3-dependent checkpoint machinery. To further investigate if SCF/Skp1 functions in resolution of recombination intermediates in cooperation with the DNA helicase activity of Fbh1, we created the *fbh1-P15A L26A* [30] and *fbh1-L14A P15A* [28] mutants carrying the dysfunctional F-box domain, which is required for the interaction to Skp1, and the *fbh1-D485N* mutant defective in the helicase activity [28] (Fig. 5E). The *fbh1-P15A L26A* mutant showed sensitivity to genotoxins during vegetative growth as the *fbh1* disruptant did (Fig. 5F) [30], whilst the *fbh1-L14A P15A* mutant showed only minor sensitivity (Fig. 5F) [28]. It is possible that the F-box function in the latter mutant might not be fully deteriorated. Reflecting the genotoxin sensitivity, the *fbh1-P15A L26A* mutant showed the bent spindle, whereas the *fbh1-L14A P15A* mutant did not (Fig. 5G). These results together indicate that the bent spindle emerges when the function of the F-box in Fbh1 is fully inhibited. The *fbh1-D485N* mutant also frequently displayed the bent spindle, confirming the necessity of helicase activity (Fig. 5G). Taken together, we conclude that not only the helicase activity of Fbh1, but also its binding to Skp1 is required for the resolution of the chromosomal entanglement. Thus, SCF/Skp1, together with the Fbh1 DNA helicase, is responsible to resolve the recombination intermediates in meiosis.

## Discussion

This study has illuminated the mechanism how the bent spindle is generated in the *skp1* mutant, clarifying the function of SCF/Skp1 in fission yeast meiosis. The bent spindle in *skp1-a7* is seen during anaphase of mitosis and MI. The spindle starts to bend before reaching the cell cortex. The nucleus containing the bent spindle neither elongate nor divide, indicating that abnormal tension is generated against microtubule extension in anaphase. It has been speculated that this is due to some defects in microtubule organisation, and/or in elasticity of the nuclear envelope. Our results shown here, however, demonstrate that the bent spindle is generated mainly by chromosomal entanglement, rather than the defects in the spindle or nuclear envelope, at least in MI. Telomeres failed to segregate, whilst kinetochores did segregate, supporting this notion. The bent spindle was no longer seen in the double mutants of *skp1-a7 rec8*Δ, indicating that the abnormal spindle tension caused by *skp1-a7* mutation is due to the chromosomal junction. Moreover, the bent-spindle phenotype was also suppressed by removal of Rec12, strongly indicating that the entanglement was generated through meiotic recombination. Indeed, Rhp51 and Rad22 foci, which localize to DSB sites during meiotic recombination in prophase, persisted even in MI, supporting the notion that the DSB is not fully repaired in the *skp1-a7* mutant. An accumulation of Rad22 and Rhp51 foci indicates that the ssDNA-containing recombination intermediates may not be resolved. Alternatively, DNA repair might be blocked in the *skp1-a7* mutant at an earlier stage before proceeding to an intermediate that can be resolved. These two possibilities are not mutually exclusive. At least in budding yeast, the DNA damage response blocks induction of gene expression necessary for the resolution of recombination intermediates [31], [32]. Thus, a failure to repair some breaks could cause a regulatory block to intermediate resolution at other breaks. This might explain why the bent spindle in the *skp1-a7* mutant was suppressed by *rad3*Δ in the mitotic cell cycle [13]. In contrast, the aberrant spindle of *skp1-a7* cells in MII was not suppressed by *rad3*Δ (Fig. 5D), indicating that in addition to the Rad3-dependent DNA damage checkpoint, some other mechanism specifically equipped for meiotic recombination may operate to block the function of repair machinery. It may also be possible that the Rad3-dependent checkpoint is not responsible for the bent spindle in meiosis.

We screened for an F-box mutant that could generate bent spindles as in the *skp1-a7* mutant, and identified Fbh1. Recently it is reported that Fbh1 is involved in the resolution of meiotic recombination [29]. This is, therefore, consistent with our results, and we further propose that SCF/Skp1 together with the F-box protein Fbh1, is involved in the repair of DSBs generated by Rec12 for meiotic recombination. We speculate that the recombination intermediates remain in the *skp1-a7* and *fbh1*Δ mutants even in MI, which results in the entanglement of chromosomes and generation of abnormal tension against the spindle. There are two DNA helicases implicated in the processing of recombination intermediates in yeast, namely Srs2 and Rqh1/RecQ [33], [34]. In *S. pombe*, *srs2*Δ cells do not show significant defects in meiosis [35]. The *rqh1*Δ mutant did not show the bent spindle in MI, in contrast to the *fbh1*Δ mutant (our unpublished results). This is consistent with the previous study reporting that Rqh1 does not play a major role in DSB formation and repair [35]. As indicated in the previous report [11], Srs2 or Rqh1 may repress accumulation of spontaneously arising recombination intermediates during the mitotic cell cycle, and Fbh1 is required for the resolution of both mitotic and meiotic recombination intermediates. Hence three DNA helicases in fission yeast play distinct roles in DNA recombination during both mitosis and meiosis.

We also found that the F-box mutant *fbh1-P15A L26A* showed the bent spindle phenotype as *fbh1*Δ and *skp1-a7* did, indicating that Skp1 and Fbh1 act together to resolve the meiotic recombination intermediates. It remains unclear, however, what biological benefit Fbh1 accepts by acting together with SCF/Skp1. It is previously reported that the F-box of Fbh1 is required for the localization of Fbh1 itself and Skp1 to the sites damaged by genotoxins [30]. We now envision that some proteins involved in recombination repair may need to be degraded through SCF-dependent proteolysis. It would be interesting to investigate the protein stability and ubiquitilation of a number of recombination repair factors, to identify the crucial substrates of SCF/Skp1-Fbh1, which would give us the new molecular insight as to how recombination intermediates are resolved in mitosis and meiosis.

## Materials and Methods

### Yeast Strains, Plasmids and Media

*S. pombe* strains used in this study are listed in Table 1. Standard methods for yeast genetics were used [36]. Briefly, for vegetative growth, a rich medium YEA, a synthetic medium SD, and a minimal medium MM with a nitrogen source were used. For induction of meiosis, *h* ^90^ heterothallic strains were plated onto the sporulation agar (SPA) or SSA and incubated at the indicated temperature (25°C–33°C) for 23∼26 hours to count the number of nuclei and/or spores, or mainly 12∼17 hours for subsequent live-cell imaging. For overexpression of the *fbh1* gene, the plasmid pREP1 [37] was used as the vector. Overexpression was induced on the minimal media without thiamine. The *rhp51-ECFP* strain is a gift from Y. Tsutsui and H. Iwasaki [38]. The GFP-Atb2 strain used in this study is previously described [39]. For construction of strains expressing other fluorescent protein tags from the native promoter and gene disruptants, the conventional methods for the PCR-based gene targeting were used [39], [40].

**Table 1:** The *S. pombe* strains used in this study.

Strain	Genotype	Figures
JY3	*h* ^90^	1A
SO1650	*h* ^90^ *skp1-a7*	1A
SO657	*h* ^90^ *GFP-atb2-kan ade6-M216 leu1-32 ura4-D18*	1B, 1C, 1D, 3B, 5B, 5C, 5D
SO656	*h* ^90^ *skp1-a7 GFP-atb2-kan ade6-M216 leu1-32 ura4-D18*	1B, 1C, 1D, 3B, 5B, 5C, 5D
SO974	*h* ^90^ *htb1-CFP-kan GFP-atb2-kan ade6-M216 leu1-32 ura4-D18*	2A
SO976	*h* ^90^ *skp1-a7 htb1-CFP-kan GFP-atb2-kan ade6-M216 leu1-32*	2A
SO1006	*h* ^90^ *mis6-2mCherry-hph GFP-atb2-kan ade6-M216 leu1-32 ura4-D18*	2B
SO1004	*h* ^90^ *skp1-a7 mis6-2mCherry-hph GFP-atb2-kan ade6-M216 leu1-32 ura4-D18*	2B
SO1043	*h* ^90^ *taz1-2mCh-hph GFP-atb2-kan ade6-M216 leu1-32 ura4-D18*	2C
SO1041	*h* ^90^ *skp1-a7 taz1-2mCh-hph GFP-atb2-kan ade6-M216 leu1-32 ura4-D18*	2C
SO1345	*h* ^90^ *rec12::ura4^+^ taz1-2mCh-hph GFP-atb2-kan ade6-M216 leu1-32 ura4-D18*	3A
SO1343	*h* ^90^ *skp1-a7 rec12::ura4^+^ taz1-2mCh-hph GFP-atb2-kan ade6-M216 leu1-32 ura4-D18*	3A
SO1129	*h* ^90^ *rec8::ura4^+^ taz1-2mCh-hph GFP-atb2-kan ade6-M216 leu1-32 ura4-D18*	3A
SO1127	*h* ^90^ *skp1-a7 rec8::ura4^+^ taz1-2mCh-hph GFP-atb2-kan ade6-M216 leu1-32 ura4-D18*	3A
SO1056	*h* ^90^ *rec12::hph GFP-atb2-kan ade6-M216 leu1-32 ura4-D18*	3B
SO1054	*h* ^90^ *skp1-a7 rec12::hph GFP-atb2-kan ade6-M216 leu1-32 ura4-D18*	3B
SO1367	*h* ^90^ *rec12::hph moa1::bsd GFP-atb2-kan ade6-M216 leu1-32 ura4-D18*	3C
SO1365	*h* ^90^ *skp1-a7 rec12::hph moa1::bsd GFP-atb2-kan ade6-M216 leu1-32 ura4-D18*	3C
SO1529	*h* ^90^ *rhp51-ECFP-ura4^+^-rhp51^+^ GFP-atb2-kan ade6-M216 leu1-32 ura4-D18*	3D
SO1527	*h* ^90^ *skp1-a7 rhp51-ECFP-ura4^+^-rhp51^+^ GFP-atb2-kan ade6-M216 leu1-32 ura4-D18*	3D
SO1514	*h* ^90^ *rad22-mCh-hph GFP-atb2-kan ade6-M216 leu1-32 ura4-D18*	3E
SO1512	*h* ^90^ *skp1-a7 rad22-mCh-hph GFP-atb2-kan ade6-M216 leu1-32 ura4-D18*	3E
SO1393	*h* ^90^ *fbh1::bsd GFP-atb2-kan ade6-M216 leu1-32 ura4-D18*	4A, 5C, 5D
SO1569	*h* ^90^ *fbh1::bsd taz1-2mCh-hph GFP-atb2-kan ade6-M216 leu1-32 ura4-D18*	4B
SO1658	*h* ^90^ *fbh1::bsd rad22-mCh-hph GFP-atb2-kan ade6-M216 leu1-32 ura4-D18*	4C
SO1593	*h* ^90^ *ade6-M216 leu1-32 ura4-D18 pREP1*	5A
SO1595	*h* ^90^ *ade6-M216 leu1-32 ura4-D18 pREP1-fbh1*	5A
SO1605	*h* ^90^ *skp1-a7 ade6-M216 leu1-32 ura4-D18 pREP1*	5A
SO1553	*h* ^90^ *skp1-a7 ade6-M216 leu1-32 ura4-D18 pREP1-fbh1*	5A
SO920	*h* ^90^ *rad3::hph GFP-atb2-kan ade6-M216 leu1-32 ura4-D18*	5B, 5D
SO922	*h* ^90^ *skp1-a7 rad3::hph GFP-atb2-kan ade6-M216 leu1-32 ura4-D18*	5B, 5C, 5D
JY878	*h* ^90^ *ade6-M216 leu1-32 ura4-D18*	5B, 5F
SO918	*h* ^90^ *rad3::hph ade6-M216 leu1-32 ura4-D18*	5B
SO1316	*h* ^90^ *fbh1::bsd ade6-M216 leu1-32 ura4-D18*	5B, 5F
SO1652	*h* ^90^ *fbh1::bsd rad3::hph ade6-M216 leu1-32 ura4-D18*	5B
SO1644	*h* ^90^ *fbh1::bsd rad3::hph GFP-atb2-kan ade6-M216 leu1-32 ura4-D18*	5C, 5D
SO1813	*h* ^90^ *fbh1-D485N ade6-M216 leu1-32 ura4-D18*	5F
SO1877	*h* ^90^ *fbh1-L14AP15A ade6-M216 leu1-32 ura4-D18*	5F
SO1891	*h* ^90^ *fbh1-P15AL26A ade6-M216 leu1-32 ura4-D18*	5F
SO1853	*h* ^90^ *fbh1-D485N ade6-M216 leu1-32 ura4-D18 pREP81-GFP-atb2*	5G
SO1886	*h* ^90^ *fbh1-L14AP15A ade6-M216 leu1-32 ura4-D18 pREP81-GFP-atb2*	5G
SO1898	*h* ^90^ *fbh1-P15AL26A ade6-M216 leu1-32 ura4-D18 pREP81-GFP-atb2*	5G

The original strain for *rhp51-ECFP* is a gift from Y. Tsutsui and H. Iwasaki.

### Microscopy

Living cells were observed in Edinburgh minimal medium without a nitrogen source for meiosis. Images in Fig. 1A were taken using Zeiss AxioplanII microscope. Other images were taken using a microscope (IX71, Olympus) with a DeltaVision-SoftWoRx system (Applied Precision) at 25°C, 30°C or 33°C. Z sectioning was done with 0.4 µm intervals, and images were taken every 1∼3 min. Images were then deconvolved, and a Z-stack projection was created.
